# The History of Durable Left Ventricular Assist Devices and Comparison of Outcomes: HeartWare, HeartMate II, HeartMate 3, and the Future of Mechanical Circulatory Support

**DOI:** 10.3390/jcm11072022

**Published:** 2022-04-05

**Authors:** Cecilia Berardi, Claudio A. Bravo, Song Li, Maziar Khorsandi, Jeffrey E. Keenan, Jonathan Auld, Sunny Rockom, Jennifer A. Beckman, Claudius Mahr

**Affiliations:** 1 Division of Cardiovascular Medicine, Baystate Medical Center, Springfield, MA 01199, USA; ceci.berardi@gmail.com; 2 Division of Cardiology, Department of Medicine, University of Washington, Seattle, WA 98195, USA; cbravo11@uw.edu (C.A.B.); lisong@uw.edu (S.L.); auldja@uw.edu (J.A.); srockom@uw.edu (S.R.); jabeckma@uw.edu (J.A.B.); 3 Division of Cardiothoracic Surgery, Department of Surgery, University of Washington, Seattle, WA 98195, USA; mkhors@uw.edu (M.K.); jekeenan@uw.edu (J.E.K.)

**Keywords:** LVAD, mechanical support, heart failure

## Abstract

The utilization of left ventricular assist devices (LVADs) in end-stage heart failure has doubled in the past ten years and is bound to continue to increase. Since the first of these devices was approved in 1994, the technology has changed tremendously, and so has the medical and surgical management of these patients. In this review, we discuss the history of LVADs, evaluating survival and complications over time. We also aim to discuss practical aspects of the medical and surgical management of LVAD patients and future directions for outcome improvement in this population.

## 1. History and Survival Outcomes

Between 2013 and 2016, the prevalence of heart failure in the United States of America (USA) was estimated to be 6.2 million and trending upward [1]. While advancements in medical therapy led to a decrease in heart failure mortality in prior years, recent trends have plateaued [1].

When considering the advanced heart failure population—a subgroup of patients with markers of severe disease who carry a particularly elevated mortality risk—heart transplantation was regarded as the only therapy with a meaningful impact on survival until recent years. Nevertheless, transplantation presents a number of challenges and limitations. In particular, the limited number of organs available and wait list mortality galvanized research into mechanical support as an alternative strategy to improve outcomes in advanced heart failure. The initial goal was to create mechanical pumps able to assist the left ventricle in its function of pumping blood to the aorta, avoiding major thrombosis, thereby allowing patients to survive in the outpatient setting until a donor heart became available. In order to do this, the device needed to be implantable in the patient’s chest, portable, and electrically powered. In 1994, the first pneumatically driven left ventricular assist device (LVAD) was approved as a bridge to transplantation (BTT) by the Food and Drug Administration (FDA) [2]. A few years later, a new indication for this type of mechanical support was explored in the Randomized Evaluation of Mechanical Assistance for the Treatment of Congestive Heart Failure (REMATCH) trial, where a vented electric LVAD was shown to improve survival compared to medical therapy in patients with advanced heart failure who were not candidates for heart transplantation [3]. These data led to the approval of the HeartMate VE LVAD device for destination therapy (DT). A technically improved version of the first HeartMate device, the HeartMate XVE, was shown to further improve outcomes in DT patients (61% survival at 1 year vs. 52% for REMATCH) [4,5]. Despite the overall survival benefit, about half of the patients implanted with this first-generation pulsatile device experienced significant adverse events, namely, infection, neurologic dysfunction, or pump failure [4].

### 1.1. Transition to Continuous-Flow Devices

The large size and limited mechanical durability of the initial pulsatile LVADs led to the development of a new generation of devices leveraging continuous-flow, rotary-pump technology. These pumps were much smaller and could therefore be utilized in smaller-sized patients; in addition, they had a single internal rotor, greatly limiting the number of moving parts subject to “wear and tear”. In a prospective observational study, continuous-flow LVADs were implanted as a bridge to transplant in 133 patients (about 20% were female) and provided effective hemodynamic support (survival was 71% at 6 months, 68% at 12 months) [6]. Soon after, a trial comparing pulsatile devices with the newer continuous-flow devices in the DT population showed the superiority of the latter in both durability and neurological outcomes [7]. Further improvements in survival were observed with the growing expertise on both surgical technique and pre- and post-operative management of these patients [8].

Despite the increase in durability and survival and the reduction in severe adverse effects, axial pumps continued to carry significant morbidity and mortality compared to heart transplantation.

### 1.2. Third-Generation Devices

**Figure 1:**
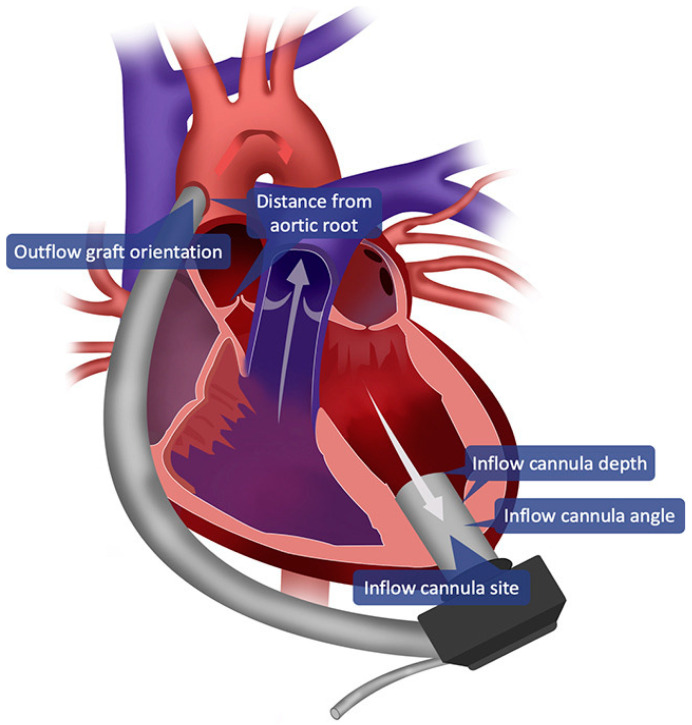
Left ventricular assist device configuration.

The third-generation devices succeeded in further reducing friction to minimize thrombosis within the continuous-flow pump and in further reducing size to allow for minimally invasive implantation techniques. The contemporary LVAD pumps, HeartMate 3 and HeartWare Ventricular Assist Device (HVAD), are intra-pericardial, directly implanted in the left ventricle (Figure 1). While the HVAD has a centrifugal impeller that utilizes a hybrid magnetic/hydrodynamic impeller suspension technology to minimize friction, the HeartMate 3 is fully magnetically levitated.

Advances in the configuration and technology related to these new-generation devices were reflected in further improvements in outcomes. In a multicenter prospective study of 140 patients eligible for transplant, HVAD was found to be non-inferior to a contemporary cohort of BTT patients from the Interagency Registry for Mechanical Assisted Circulatory Support (INTERMACS) database (the estimated 180-day survival was 94%) [9]. A follow-up study including 192 additional patients who were implanted with HVAD in the continuous access protocol showed similar results (survival at 180 days was 91%) [10].

The Multicenter Study of MagLev Technology in Patients Undergoing Mechanical Circulatory Support Therapy with HeartMate 3 (MOMENTUM 3) trial compared the outcomes of BTT and DT patients randomized to the new centrifugal pump (HeartMate 3) versus axial pumps (HeartMate II) [11]. Among 366 patients, the rate of survival free of disabling stroke or survival free of reoperation was 77.9% in the HeartMate 3 group and only 56.4% in the HeartMate II group during the 2-year follow-up period. Importantly, pump thrombosis was exceedingly rare with the new device, and ischemic stroke was significantly less frequent than with the HeartMate II. Since the completion of this trial, the device manufacturer subsequently made the decision to discontinue production of the HVAD, which means that, at this point, the only FDA-approved durable LVAD is the HeartMate 3.

### 1.3. Real-Life Outcomes from Observational Studies

In the INTERMACS registry, the survival of patients with LVADs has improved over time. In a recently published report, the 1-year survival rate for patients implanted between 2015 and 2019 was 86.8% for BTT and 80.1% for DT [12]. These percentages are higher than those observed for patients implanted between 2010 and 2014, and the same trend is seen in the 2-year survival data. Despite the increasing acuity in this population and the higher number of DT LVADs implanted in recent years, the median survival time has increased from 46.5 months (95% CI: 44.7%, 48.2% months) in the previous era to 54.6 months (95% CI: 52.1%, 58.2% months) in the current era.

A gap still exists between LVAD and heart transplant survival outcomes. In fact, 1-year mortality post-transplant is about 10% [13]. As mentioned before, transplant hearts are a very limited resource; therefore, there are stricter eligibility criteria. In addition, the wait time once listed is often months; this seemingly brief time should be contextualized within the elevated morbidity and mortality related to advanced heart failure.

Overall, treatment of end-stage HF with an LVAD consistently improves patient quality of life [14,15]. After receiving LVAD support, patients typically experience improved functional capacity with lesser physical and psychological symptoms, leading to better quality of life [16,17,18]. In addition, psychosocial factors have been shown to significantly influence quality of life after LVAD implant. Strong social support from caregivers, family, and friends is associated with better quality of life, while more severe depression appears to reduce improvement in quality of life after LVAD implantation [19,20]. Although cognitive function typically improves after LVAD therapy [21,22], patients with continued cognitive dysfunction tend to have poorer quality of life [23].

It is important to acknowledge that LVAD patients continue to present a high rate of hospitalization (more than 30% at 90 days and more than 70% at 12 months) [12]. This is mostly due to the frequent incidence of adverse events. The prevalence of severe events such as pump thrombosis, pump failure, or severe post-operative bleeding has decreased over time due to advancements in pump technology, patient selection, and surgical technique. Unfortunately, infection, right ventricular failure, stroke, and gastrointestinal bleeding remain a relatively frequent occurrence in the LVAD population and have a negative impact on the patients’ quality of life [12].

## 2. Patient Management and Optimization

The main goal of the team caring for LVAD patients is to obtain longer survival while minimizing adverse events. In order to achieve this goal, the following need to be addressed and optimized: (1) patient selection, (2) surgical management, and (3) medical management for the prevention and treatment of adverse events.

### 2.1. Patient Selection

The International Society of Heart and Lung Transplantation (ISHLT) recently issued updated guidelines regarding the selection of appropriate candidates for durable mechanical support devices [24]. Evaluation prior to LVAD implantation includes (1) cardiac evaluation, (2) careful adjudication of the patient’s non-cardiac co-morbidities, and (3) comprehensive evaluation of the patient’s social support and psychosocial risk factors. The goal of this extensive workup is to identify those stage D heart failure patients with reduced ejection fraction who will derive a benefit from the device in terms of both survival and quality of life.

Given the intrinsic high mortality associated with advanced heart failure, the timing of LVAD implantation and pre-operative optimization are crucial. Risk stratification tools such as the Seattle Heart Failure Model may help decision making in this setting [25,26]. In addition, shared decision making with the patient and their caregivers is key. This involves in-person education, carried out by physicians and LVAD coordinators, as well as reading material, videos, or the use of shared decision-making tools.

#### 2.1.1. Medical Evaluation

In order to select the appropriate candidates to undergo LVAD implantation, an extensive evaluation is carried out. Tests are conducted to confirm indications and rule out contraindications. Each system needs to be assessed and discussed in a multidisciplinary forum in order to devise an individualized plan for the patient. Key components of the pre-operative evaluation are summarized in Table 1.

#### 2.1.2. Psychosocial Considerations in Patient Selection and Quality of Life

Psychosocial considerations are critical to the selection and management of LVAD patients. Current guidelines for mechanical circulatory support recommend assessing psychosocial factors including social support, mental illness, cognitive function, substance abuse, adherence behaviors, health literacy, housing, and financial support [24,27,28]. Psychosocial evaluations are conducted by social workers, often using standardized modified transplant psychosocial evaluation tools like the Stanford Integrated Psychosocial Assessment of Transplant (SIPAT), and reviewed by a multidisciplinary team [29].

While recent evidence indicates that there is a limited impact of psychosocial factors on survival after LVAD implantation, their impact on readmission and adverse events has been consistently shown [29,30,31,32]. For example, DeFilippis et al., using INTERMACS Registry data from 15,403 LVAD recipients, reported that the presence of at least one psychosocial risk factor (limited social support, limited cognition, substance abuse, severe psychiatric disease, or repeated non-compliance) was associated with device-related adverse events such as infections, gastrointestinal bleeding, or pump thrombosis and with a reduced likelihood of receiving heart transplantation, but did not lead to a lower survival rate [30]. This study, as well as others showing psychosocial factors influencing readmission rates and adverse events [29,31,32,33,34], supports the importance of evaluating psychosocial factors to assess LVAD candidacy and to plan post-implant care to optimize patients for heart transplant or life-long LVAD therapy.

Some psychosocial factors are essential for LVAD candidacy. Patients must have a dwelling with adequate access to power to operate the LVAD equipment safely, sufficient cognitive function to operate and manage the pump, health coverage to cover costs of life-saving medications such as anticoagulants that prevent pump thrombosis, and a caregiver who can support the LVAD patient during the transition from the hospital to home care with vital caregiving duties (e.g., sterile dressing changes, bathing, transportation to clinic) [24,27]. The absence of one or more of these requirements typically precludes LVAD implantation as the risk for an adverse outcome is considered excessively high compared to continued medical management without LVAD therapy.

The influence of additional psychosocial factors like health literacy, mental illness, adherence behaviors, and substance abuse is more subjective. Each of these factors has been shown to increase the risk of readmission after implant or has been associated with an increased risk of having an adverse LVAD event [30,35,36]. Although these factors do not disqualify an individual from receiving an LVAD, their presence and severity are evaluated carefully to determine whether LVAD implantation is likely to be more beneficial than harmful, and to plan appropriate care both prior to and after the LVAD implantation. With the cooperation of providers and patients, many psychosocial risk factors can be managed with individualized plans that support the patients in reaching their goals, including transplant, better functional status, and improved quality of life [36]. An additional consideration is that drug and alcohol rehabilitation or other specialty care centers (i.e., psychiatric) may not accept patients with LVADs. Thus, patients needing those services may not be effectively managed after the implant [37]. A thorough psychosocial assessment provides critical information to inform appropriate LVAD candidacy and to create effective plans of care that can significantly improve the LVAD patient’s quality of life. Additional research and increased transparency in how implanting centers make decisions to provide LVAD therapy are needed to elucidate the role and impact of psychosocial risk factors in determining LVAD candidacy.

### 2.2. Surgical Management

#### 2.2.1. General Considerations for LVAD Configuration

The two most common approaches for LVAD implantation are median sternotomy and a bi-thoracotomy approach. Methods of LVAD implantation without the use of cardiopulmonary bypass have been described, but they are not widely adopted [38,39]. In general, the utilization of cardiopulmonary bypass provides effective control of the circulation during the procedure and allows for decompression and opening of the left ventricle (LV) during the period of pump implantation. It also allows for safe de-airing of the heart and pump once the pump is implanted.

The key objectives of LVAD implantation include (1) establishing an unobstructed inflow cannula position in the left ventricle and (2) properly positioning the outflow graft anastomosis without obstruction or kinking. The only currently available FDA-approved LVAD, the HeartMate3, like the historical HVAD and HeartMate II devices, was designed to pass an inflow cannula through the apex of the heart. This cannula is ideally directed toward the mitral valve in order to optimally drain blood from the LV through the outflow graft into systemic circulation. Misalignment of the inflow cannula can result in thrombotic events due to a greater propensity for areas of blood stagnation within the LV. The optimal site for inflow position is usually around the apex of the LV.

The usual site for outflow graft anastomosis is the ascending aorta, although alternative sites such as the axillary artery or descending aorta have also been utilized. In general, the outflow graft should be sized such that it can travel across the midline along the inferior margin of the heart before gently bending cranially to travel along the right atrium to the right of the sternal midline to then finally reach the ascending aorta (Figure 1). Excessive length on the outflow graft can be problematic due to the potential for kinking, and there is some thought that this may also lead to increased risk of outflow graft thrombotic complications. Conversely, if the outflow graft is too short, tenting and stenosis may result, and the graft will be more likely to underlie the midline to a greater degree, complicating re-entry for future reoperations.

#### 2.2.2. Considerations for Destination Therapy versus Bridge to Transplant LVAD Implantation

The general principles that must be achieved are the same for DT or BTT LVAD implantation. However, given that a second operation is necessarily planned for BTT patients, there are some special considerations with BTT implantation, so as to reduce the risk of re-entry injury at the time of transplantation. The conventional approach to LVAD implantation has been through median sternotomy. However, the bi-thoracotomy approach has also been popularized in many centers. There are pros and cons associated with each technique [40,41,42].

The median sternotomy approach is the most familiar incision for cardiac surgeons. It allows for the best possible surgical exposure. Usually, there is minimal postoperative pain with this approach, as compared to other ports of access to the chest. It allows for ease of optimal placement of the inflow cannula and outflow graft.

The less invasive approach includes a bi-thoracotomy incision in the left lateral chest wall over the left ventricular apex area to place the inflow cannula and in the right second intercostal space to place the outflow graft [40,41,42]. The role of this less invasive approach is primarily in BTT patients, and the main advantage is preservation of the sternum for the subsequent heart transplantation. There are also reports of a lower risk of bleeding and right ventricular failure with this approach. The disadvantages include the need for peripheral cannulation for cardiopulmonary bypass and limited exposure potentially leading to suboptimal positioning of the inflow cannula and outflow graft onto the LV and the ascending aorta, respectively.

### 2.3. Medical Management to Minimize Adverse Events

#### 2.3.1. Stroke

Ischemic and hemorrhagic strokes are a relatively common LVAD complication that occurs at a rate of 0.08 to 0.27 events per patient per year [43]. In this patient population, a stroke can be devastating and is associated with worse outcomes and lower chance of receiving a heart transplant within the subgroup of BTT patients [44,45].

LVAD patients often present the classic cardiovascular risk factors associated with a higher risk of stroke. However, other LVAD-specific factors have been reported to further increase this risk. For instance, ischemic strokes in LVAD patients originate not only from atherosclerotic disease, but also from embolization of thrombi formed in the LVAD itself, outflow graft, intracardiac chambers, or aortic root [43,46,47]. Therefore, optimizing the LVAD speed to allow for intermittent aortic valve opening and maintaining therapeutic anticoagulation in addition to antiplatelet therapy are key to reducing thrombus formation.

At the same time, LVAD patients are prone to bleeding due to the need for anticoagulation and antiplatelet therapy, but also due to acquired deficiency of Von Willebrand factor, secondary to shear stress from continuous flow. Intracranial bleeding in LVAD patients can be spontaneous or due to hemorrhagic transformation of an ischemic stroke, or septic emboli, among other mechanisms.

Additionally, there is a clear association between the risk of stroke and device type. The HM3 has been shown to be associated with lower ischemic stroke risk than the HMII [48,49]. Blood pressure plays an important role in determining this risk. In fact, maintaining a mean arterial pressure (MAP) of ≤85 mmHg measured by the Doppler opening pressure in patients with the HVAD device reduced the stroke risk to a rate similar to that in the HMII population [50]. Given the importance of blood pressure control to prevent adverse events, our program adheres to a strict blood pressure protocol. Blood pressure is obtained via manual cuff and a Doppler: the Doppler opening pressure sound approximates the mean arterial pressure in the majority of patients, up to a pulse pressure of about 30 mmHg. An ideal Doppler opening pressure target for LVAD patients is 70 mmHg, with an acceptable range of 60–80 mmHg. If there is a concern of a possible discrepancy between the Doppler opening pressure and mean arterial pressure, we conduct a formal arterial line study. During this, an arterial line is placed and multiple invasive MAP measurements and Doppler opening pressures are obtained from the ipsilateral arm for comparison. If the patient is hypertensive by invasive mean arterial pressure, they are administered hydralazine to lower their arterial pressure to the target range. Successive Doppler readings are then compared to the MAP under these ideal circumstances. If a significant discrepancy exists between the invasive mean arterial pressure and Doppler opening pressure, we document that difference and adjust the Doppler opening pressure goal accordingly.

#### 2.3.2. Right Ventricular Failure

Unlike the left ventricle, the right ventricle (RV) is a highly compliant, thin-walled, crescent-shaped chamber that usually works in a low-pressure, low-resistance system. Around 10 to 40% of people who receive an LVAD develop right ventricular failure early during the post-implantation period, leading to poor outcomes [51,52,53]. Several factors play a role in the development of this complication, including baseline right ventricular function, increased right ventricular loading conditions, and changes in cardiac geometry that can lead to worsening tricuspid regurgitation or affect the inter-ventricular septum contribution to right ventricular function [52,54,55,56]. Additionally, changes in pulmonary vascular resistance and arrhythmias can further contribute to the development of right ventricular failure.

Although at this point there are no proven medical therapies available to prevent or treat right ventricular failure, there are several interventions that can provide adequate support while a more favorable hemodynamic status is re-established. These include inotropic or temporary mechanical circulatory support of the right ventricle, pulmonary vasodilators such as phosphodiesterase inhibitors, volume optimization, and hemodynamic and echocardiographic LVAD speed optimization targeting minimal mitral regurgitation, to avoid displacing the septum excessively into the left ventricle [57]. Preserving the pericardial structure by performing the LVAD implantation via lateral thoracotomy has also been associated with lower incidence of right ventricular failure [58]. Finally, avoiding ventricular arrhythmias and maintaining sinus rhythm might also positively affect the right ventricular function. The majority of late RV failure is due to residual hypervolemia, sub-optimal blood pressure control, or chronic excess VAD speed. Thus, it is imperative to ensure that VAD patients are diuresed to euvolemia, have strict blood pressure control, and have optimized VAD speed to avoid iatrogenic RV pressure/volume overload from excess VAD speed.

#### 2.3.3. Gastrointestinal Bleeding

After infectious complications, gastrointestinal (GI) bleeding is the most common problem seen in LVAD patients, affecting up to 40% of this patient population [59]. There are several LVAD-related elements that contribute to the pathogenesis of bleeding complications, beyond the use of chronic anticoagulation and antiplatelet therapy. The shear stress generated when the blood is propelled through the LVAD causes degradation of Von Willebrand factor [60]. The same shear stress stimulates proangiogenic factors that lead to angiogenesis and the formation of intestinal arterio-venous malformations [61]. If the patient has concomitant right ventricular failure, elevated right-sided pressures increase venous congestion, raising the bleeding risk from pre-formed arteriovenous malformations [62,63]. Several interventions have been proposed to help decrease the incidence of gastrointestinal bleeding; however, none of them have been proven effective in large trials. Maintaining a stable international normalized ratio (INR), particularly avoiding supratherapeutic values, may help prevent this complication. Medications that have been reported to be associated with lowering the risk of gastrointestinal bleeding include octreotide [64], thalidomide [65], and omega-3 [66], among others.

In the setting of gastrointestinal bleeding, the treatment goal is to ensure hemodynamic stability by providing transfusions as needed and, in selected cases, by partially reversing the INR while considering the risk of LVAD thrombosis. Esophagogastroduodenoendoscopy (EGD), colonoscopy, and video capsule endoscopy can provide essential information to rule out other etiologies of gastrointestinal bleeding and to guide therapy. Endoscopy allows for direct intervention on the arteriovenous malformations that are most frequently the cause of GI bleeding.

The usual target INR for LVAD patients is 2.5, with a range of 2–3 [67]. A subtherapeutic INR requires bridging in our institution, using unfractionated heparin in an anti-Xa based fashion, targeting 0.3–0.5 until the INR becomes >2. As most bleeding complications are not usually life-threatening, it is our programmatic approach to only fully reverse therapeutic anticoagulation for life-threatening, usually central nervous system bleeding events.

#### 2.3.4. Driveline Infections

Driveline exit site infections are among the most common complications observed in LVAD patients [68]. Risk factors for this complication are related to patient characteristics and co-morbidities, device type, and specific events, like driveline trauma.

Some examples of patient characteristics that increase the risk of driveline infections are obesity, younger age, worse constitution, and longer time on device support [69]. Co-morbidities like diabetes, chronic kidney disease, and depression have also been associated with higher risk of driveline infection [69]. A single-center analysis showed that driveline infections are more common among HM3 patients, possibly due to the bigger size of the driveline in this device [70].

Strategies to prevent driveline infections include ensuring that the velour part of the driveline is entirely within the tissue tunnel, using fixation anchors to maintain optimal driveline position, having a standardized dressing change protocol with strict sterile technique, and patient education [69].

Once a driveline infection is diagnosed, treatment depends on the organism isolated and on characteristics of the host. In the majority of cases, chronic suppressive antibiotic therapy is needed due to an inherent inability to achieve source control. As many organisms are biofilm producing, curative antibiotic therapy is not feasible. The only definitive therapy is LVAD explantation (recovery or transplant).

## 3. Future Directions

Despite tremendous advances in technology and improved outcomes over the last two decades, VAD therapy remains far from optimal for end-stage heart failure patients. Currently, it remains saddled by undesirable complication rates and significant quality-of-life barriers. The future of VAD therapy should aim to achieve the same long-term survival rates as cardiac transplantation, while minimizing the negative impact on quality of life. Incremental improvements currently being developed include enhanced blood-pump interface, fluid dynamics, and impeller engineering to improve hemocompatibility and reliability. More revolutionary goals include a ‘smart’ VAD that modulates pump flow to adapt to dynamic physiological needs and perhaps simulates more natural pulsatile flow synchronized with the patient’s native heart residual contractility. Early developments of such a dynamic VAD in simulations and animal models have been published, and the results are encouraging [71,72,73,74,75]. Another goal that can significantly improve quality of life and decrease infection risk is the development of a fully implantable device. A fully implantable total artificial heart has been tried before with a transcutaneous energy transfer device, which was able to successfully power the device [76]. Similar technology is currently being developed for VADs.

In addition to improving the VAD hardware, the future of VAD therapy also depends on improving the software that controls and monitors VADs. New technology such as machine learning is poised to take on an important role in VAD therapy in the future. Machine learning may be used to monitor VAD operating metrics for early detection of VAD complications or to identify physiological changes such as volume status and arrhythmias. Initial proof-of-concept studies leveraging machine learning to detect VAD suction, impending pump failure, and arrhythmias have been published [77,78,79]. In a sense, the VAD is a perfect host for machine learning algorithms, as it is an implanted wearable device with a built-in computer supplied by continuous power. Coupled with reliable physiological sensors in a future VAD, machine learning and artificial intelligence have the potential to power a closed-loop, completely autonomous VAD that continuously optimizes the patient’s cardiac physiology.

With these technological enhancements, a future generation of VADs whose outcomes exceed those of cardiac transplantation is finally within reach.

## Figures and Tables

**Table 1:** Pre-operative evaluation of left ventricular assist device candidates.

	Laboratory Test	High Risk Features	Contraindications
**Cardiac Evaluation**			
	Right heart catheterization	- Elevated PVR - Elevated TPG	
	Trans-thoracic echocardiogram	- Valvular disease - Right ventricular dysfunction	
	Left heart catheterization	- Significant, multivessel, obstructive coronary disease	
	Electrocardiogram	- Refractory ventricular arrhythmia	
	Cardiopulmonary exercise test	- Significant pulmonary disease	
**Non-cardiac evaluation**			
*Renal*	Basic metabolic panel	- eGFR < 30	Hemodialysis
*Gastrointestinal*	EGD/Colonoscopy	- Significant ulcers, AVMs	Malignancy with poor 5-year survival
*Hepatic*	Liver panel, liver ultrasound If needed to rule out cirrhosis: liver biopsy, portal pressure measurement	- Chronic liver disease with bilirubin 1–3 g/dL - Acute liver injury without improvement in the 48 h prior to implant	Chronic liver disease with bilirubin > 3 g/dl, Evidence of cirrhosis MELD > 17
*Hematology*	CBC, coagulation panel, HIT panel in selected patients	- Any pro-thrombotic state	
*Oncology*	Age-appropriate screening tests	- History of prior malignancy	Active malignancy
*Vascular*	Vascular ultrasound Ankle-brachial index	- Significant lower extremity vascular disease (high risk of complications during cardiopulmonary bypass cannulation). - Ascending aorta calcifications, significant carotid plaque (increased risk of stroke)	
*Pulmonary*	Pulmonary function test Lung imaging	- Low FEV1 and FVC, DLCO < 50% of predicted - Extensive pulmonary pathology can increase risk of post-operative RV failure	
*Infectious disease*	Microbiology tests and imaging depending on the patient’s history and physical exam	- Recent treated infection, especially nosocomial	Active infection
*Endocrine*	TSH HbA1c	- Poorly controlled diabetes	
*Nutrition*	Albumin, pre-albumin	- BMI < 20, BMI > 40, albumin < 3.2 mg/dl, pre-albumin < 15 mg/dL	
*Neurologic*	CT head or MRI head Neurocognitive evaluation	- Patients with a history of prior cerebrovascular accident	Substantial neurologic deficits or neurocognitive disabilities impairing functional status
*Dental*	X-ray or CT if indicated	- Active dental infection	
**Psychosocial**			
	SIPAT score Comprehensive psychosocial and substance abuse history	- Active Substance abuse - Untreated or newly diagnosed psychiatric disease - History of noncompliance - Lack of caregiver support - Lack of insurance coverage	Poor psychosocial profile with no viable plan for improvement in a relative short timeframe, lack of stable housing, at risk of incarceration

AVMs: arteriovenous malformations; BMI: body mass index; CT: computed tomography; DLCO: diffusing capacity of the lungs for carbon monoxide; EGD: esophagosastroduedonoscopy; eGFR: estimated glomerular filtration rate; FEV: forced expiratory volume; FVC: forced vital capacity; HIT: heparin-induced thrombocytopenia; MELD: model for end-stage liver disease; MRI: magnetic resonance imaging; PVR: pulmonary vascular resistance; TPG: trans-pulmonary gradient; SIPA: Stanford Integrated Psychosocial Assessment.
